# Dr. Auguste Zundel

**Published:** 1885-10

**Authors:** 


### OBITUARY.

### DR. AUGUSTE ZUNDEL.

Dr. Auguste Zundel, the superior veterinarian of Alsace-Lorraine, died at Strasburg on June 11th. The doctor had a world-wide reputation by his numerous scientific writings in the various journals, but more particularly by his edition of De Arbeval's "Dictionaire de Medicines, de Chirurgie, et d'Hygiene Vétérinaires." He was a most indefatigable worker, and his services were not only recognized by the several veterinary societies, but by the ruling powers of several countries.

E. W. PERRY, V.S., M.D., died of apoplexia July 27, at New Bedford, Mass., where he was in practice. Dr. Perry was a graduate of Harvard Medical School, as well as of the Royal Veterinary College, London.

